# A FRAMEWORK OF SUPPORT: THE NURSING HOME PERSON-CENTERED OPTIONS COUNSELING CERTIFICATION PROGRAM

**DOI:** 10.1093/geroni/igad104.1899

**Published:** 2023-12-21

**Authors:** Kate Crary, Melissa Mandrell, Laura Davie

**Affiliations:** University of New Hampshire, Durham, New Hampshire, United States; University of New Hampshire, Durham, New Hampshire, United States; University of New Hampshire, Durham, New Hampshire, United States

## Abstract

Person-centered initiatives and approaches are the cornerstone of best practices when it comes to supporting older adults and persons living with dementia. Lack of person-centered care has been associated with higher medical costs and negative health outcomes, particularly in low-resource communities. Implementing person-centered care may help to address disparities and improve care outcomes in these communities, although it is often unclear which approaches are most appropriate. The Person-Centered Options Counseling Certification program attempts to address this gap via a mentoring program and competency framework. Developed and piloted in rural New Hampshire, this framework helps professionals and organizations evaluate the approaches that works best for them, while creating fidelity for ongoing support through a rigorous mentorship program. The framework shows promise of being cost effective, an important consideration for many low-resource communities. Professionals can attain knowledge, skills, and abilities without incurring higher education costs and agencies can utilize pre-developed instructional materials. Pilot data was collected to evaluate the program and demonstrated that professionals found the educational training delivered within the framework was relevant to their community and increased their knowledge about person-centered care, although some expressed hesitancy when applying the skills learned with individuals. Professionals who completed both the training and participated in the mentorship program reported a high level of competency, satisfaction, and confidence in delivering person-centered care. The use of this framework may support staff retention in low-resource communities and approaches can be tailored across different environments to facilitate the delivery of person-centered care.

